# Gut dysbiosis contributes to chlamydial induction of hydrosalpinx in the upper genital tract

**DOI:** 10.3389/fmicb.2023.1142283

**Published:** 2023-04-13

**Authors:** Qi Tian, Tianyuan Zhang, Luying Wang, Jingyue Ma, Xin Sun

**Affiliations:** ^1^Department of Obstetrics and Gynecology, Hunan Provincial Maternal and Child Health Care Hospital, Changsha, Hunan, China; ^2^National Health Commission Key Laboratory for Birth Defect Research and Prevention, Hunan Provincial Maternal and Child Health Care Hospital, Changsha, Hunan, China; ^3^Key Lab of Molecular Virology and Immunology, Institute Pasteur of Shanghai, Chinese Academy of Sciences, Shanghai, China; ^4^Department of Obstetrics and Gynecology, Third Xiangya Hospital, Central South University, Changsha, Hunan, China; ^5^Department of Dermatovenereology, Tianjin Medical University General Hospital, Tianjin, China

**Keywords:** Oral antibiotics, gut dysbiosis, *Chlamydia muridarum*, genital tract, pathology

## Abstract

*Chlamydia trachomatis* is one of the most common sexually infections that cause infertility, and its genital infection induces tubal adhesion and hydrosalpinx. Intravaginal *Chlamydia muridarum* infection in mice can induce hydrosalpinx in the upper genital tract and it has been used for studying *C. trachomatis* pathogenicity. DBA2/J strain mice were known to be resistant to the chlamydial induction of hydrosalpinx. In this study, we took advantage of this feature of DBA2/J mice to evaluate the role of antibiotic induced dysbiosis in chlamydial pathogenicity. Antibiotics (vancomycin and gentamicin) were orally administrated to induce dysbiosis in the gut of DBA2/J mice. The mice with or without antibiotic treatment were evaluated for gut and genital dysbiosis and then intravaginally challenged by *C. muridarum*. Chlamydial burden was tested and genital pathologies were evaluated. We found that oral antibiotics significantly enhanced chlamydial induction of genital hydrosalpinx. And the antibiotic treatment induced severe dysbiosis in the GI tract, including significantly reduced fecal DNA and increased ratios of firmicutes over bacteroidetes. The oral antibiotic did not alter chlamydial infection or microbiota in the mouse genital tracts. Our study showed that the oral antibiotics-enhanced hydrosalpinx correlated with dysbiosis in gut, providing the evidence for associating gut microbiome with chlamydial genital pathogenicity.

## Introduction

*Chlamydia trachomatis* can cause long-lasting genital fibrosis and may result in tubal infertility (Strandell et al., 1994; Vasquez et al., 1995; Land et al., 2010; Budrys et al., 2012; Jansen et al., 2016), but the mechanism of the process remains poorly understood. Mouse genital infection with *Chlamydia muridarum* was a widely used model to investigate chlamydial pathogenesis. Tubal inflammation caused by chlamydial ascension was defined as a key pathogenic factor in this model (Rodgers et al., 2010, 2011; Budrys et al., 2012; Chen et al., 2014; Lei et al., 2014; Liu et al., 2014; Yang et al., 2014; Zhong, 2017). Many chlamydial (Liu et al., 2014; Huang et al., 2015) and host (Natividad et al., 2007; Asquith et al., 2011; Li et al., 2012; Andrew et al., 2013; Dong et al., 2014; Yang et al., 2014; Manam et al., 2015) factors were identified to affect the chlamydial ascension (Chen et al., 2010; Frazer et al., 2013) and tubal inflammation (Cheng et al., 2008; Murthy et al., 2011; Yang et al., 2014). Interestingly, not all mice with *C. muridarum* ascension and tubal inflammation may develop hydrosalpinx (Chen et al., 2014). In human cases, only a portion of women with high titers of serum anti-Chlamydia antibodies develop long-term complications (Rodgers et al., 2010, 2011; Budrys et al., 2012). These observations suggested possible extra-genital mechanisms in the development of genital pathology during chlamydia infection.

Studies showed that genital microbiota played a role in the host susceptibility to chlamydial infection (Nelson et al., 2010; Ravel et al., 2011; Bai et al., 2012; Hickey et al., 2012; Ma et al., 2012; Ravel and Brotman, 2016; Noyes et al., 2018; van Houdt et al., 2018). *Lactobacillus iners* dominated genital or cervicovaginal microbiota was associated with high susceptibility to chlamydial infection while *L. crispatus* promoted resistance (Nardini et al., 2016; van Houdt et al., 2018). It is assumed that certain genital microbiota may cooperate with *C. trachomatis* (Ziklo et al., 2016) and help it to evade the inhibition of IFN-γ by taking up indole from Prevotella (Ravel et al., 2011; Ziklo et al., 2016). Although the genital microbiome is associated with chlamydial ascending infection, the causative correlation between these two remains unclear. And the role of gut microbiome in chlamydial pathogenicity has not been investigated. It has been shown that gut microbiota may regulate fibrosis process in the development of extra-gut pathologies by inducing profibrotic regulatory T cells (Metwali et al., 2006; Owyang et al., 2006; Koch and Muller, 2015; Tai et al., 2016; Shivaji, 2017). We hypothesized that gut microbiota might be able to impact chlamydial pathogenicity in the upper genital tract considering the fact that fibrosis is a key feature of chlamydial pathogenicity (Murthy et al., 2007; Chen et al., 2009; Rodgers et al., 2010, 2011; Peng et al., 2011; Budrys et al., 2012; Li et al., 2012; Lu et al., 2012; Chen et al., 2014; Lei et al., 2014; Liu et al., 2014; Tang et al., 2014; Yang et al., 2014; Yadav et al., 2017; Zhang et al., 2014; Zhong, 2017).

The DBA2/J mice were known to resist the development of hydrosalpinx following an intravaginal infection with *C. muridarum* (Chen et al., 2014). In the current study, oral antibiotic (vancomycin and gentamicin, ABX) cocktails were given to DBA2/J mice to cause gut dysbiosis and the mice were further intravaginally challenged by *C. muridarum* to induce hydrosalpinx. Since the vancomycin and gentamicin cannot bypass eukaryotic cell membrane or affect chlamydial infection (Ridgway et al., 1978; Dailloux et al., 1990), it allowed us to evaluate the effect of the ABX-induced gut dysbiosis on chlamydial pathogenicity selectively. We found that the oral ABX indeed promoted chlamydial induction of hydrosalpinx significantly without changing the chlamydial infection courses in the lower or upper genital tract. The ABX caused severe dysbiosis in the gastrointestinal (GI) tract, including significantly reduced fecal DNA and increased ratios of firmicutes over bacteroidetes but ABX did not change vaginal microbiota greatly. Furthermore, the oral antibiotics did not affect *C. muridarum* colonization in the GI tract either.

The current study has provided the experimental evidence for associating gut microbiome with chlamydial pathogenicity. This study may also provide a platform to study the impacts of gut microbiome on genital tract pathogenesis.

## Materials and methods

### The growth of chlamydial organism

The *Chlamydia muridarum* derived from Mouse Pneumonitis strain Nigg II (ATCC #VR-123) was used in this study. The *Chlamydia muridarum* organisms were grown in HeLa cells and purified as elementary bodies as previously described (Fan et al., 1998; Zhang et al., 2015). The purified EBs were stored in aliquots at -80^o^ C until use.

### Mice and treatment

DBA2/J mice (6–7 weeks old female) were purchased from Vital River, Beijing. Mice were bred and maintained under specific pathogen free (SPF) conditions in the institutional animal facility of the Institute of Pasteur of Shanghai Chinese Academy of Sciences. Mice were treated with or without a cocktail of vancomycin (A100990-0001, Sangon Biotech, Shanghai) and gentamicin (A620217-0025, Sangon Biotech, Shanghai) *via* oral gavage on days 1 and 2. The ABX cocktail was then maintained in drink water in the ABX group until mice were sacrificed. For oral gavage, vancomycin and gentamicin were used 3 mg/ml each in a total volume of 200ul per dosing per mouse. 0.5 mg/ml each of vancomycin and gentamicin were used in drinking water. Twenty-one days after the ABX treatment, mice were intravaginally challenged with 2 ×10^5^ IFUs of *Chlamydia muridarum*. Vaginal and rectal swabs were taken on days 3 and 7 post and then on a weekly basis thereafter. Specifically, on each sampling occasion, one vaginal swab and one rectal swab were obtained from each mouse for analysis. The swabs were further analyzed for live chlamydial organisms. Tissue chlamydial organisms were measured in some experiments by tissue homogenization.

### Titrating live chlamydial organisms recovered from swabs and tissue homogenates

To quantitate live chlamydial organisms in vaginal or rectal swabs, each swab was immersed in 0.5 ml of iced SPG and vortexed with glass beads in 1.5 ml tube (Chen et al., 2015). The tube was centrifuged and the chlamydial organisms in the supernatants were then titrated on HeLa cell monolayers. The infected cell cultures were further processed for immunofluorescence assay as previously described (Kapoor et al., 2023). For each titration, the inclusions were counted under a fluorescence microscope and five random fields were counted for each coverslip. The total number of IFUs per sample was calculated according to the mean IFUs per view, the ratio of the view area to the well, dilution ratio, and volumes. The total number of IFUs/swab was then converted by log10 and the final number presented the chlamydial burden of each mouse at each time point. To quantitate chlamydial organisms in tissues, the tissue (or organ) was homogenized in 0.5 to 5 ml cold SPG depending on the sizes with an automatic homogenizer [Omni Tissue Homogenizer, TH115, Kennesaw, GA]. The homogenates were sonicated and spun at 3000 rpm for 5 min. The supernatants were titrated and counted for live *C. muridarum* organisms the same as the swabs and as previously described (Lei et al., 2014).

### Immunofluorescence assay

The primary antibody: A rabbit antibody (made with purified *C. muridarum* elementary bodies) was gift from Jingyue Ma in Tianjin Medical University General Hospital. After primary antibody incubation, the secondary antibody (goat anti-rabbit IgG conjugated with Cy2, #ab6940, Abcam) was added and the nuclei was visualized by DNA dye Hoechst 33258 (#ab228550, Abcam). The doubly labeled samples were used for counting for chlamydial inclusions under a fluorescence microscope (AX70, Olympus) with a CCD camera (Hamamatsu).

### Evaluation of genital macroscopical pathology

Seventy days after *C. muridarum* infection, mice were sacrificed for evaluating genital pathology. The upper genital tract hydrosalpinx was evaluated. Before the genital tract was removed, an *in situ* gross examination was made for evidence of oviduct hydrosalpinx.

The oviduct hydrosalpinx score was made based on the following criteria: no hydrosalpinx (0 score), hydrosalpinx detectable only under microscope (1 score), hydrosalpinx visible with naked eyes but the size was smaller than the ovary on the same side (2 score), equal to the ovary on the same side (3 score) or larger than the ovary on the same side (4 score). Scores from both sides of the oviduct from the same mouse were added up to represent the score for a given mouse. The individual mouse scores were calculated into means ± standard errors.

### Fecal microbiota evaluation

Fecal samples were collected from female DBA2/J mice both before and after the oral ABX treatment. The fecal samples were frozen in liquid nitrogen and stored at -80°C until DNA extraction. The fecal genomic DNA was extracted using the QIAamp DNA Stool Mini kit (Qiagen Inc., Germantown, MD20874) according to the manufacturers’ instructions. The purity and quantity of DNA samples were determined by absorbance ratios at 260/280. A real time/quantitative PCR (iTaq universal SYBRgreen super mix 500, Bio-Rad) was used to quantitate bacterial 16S rRNA genes in each sample. The following PCR condition was used: 95°C x 5 min for denaturation, then 95°C x 5 s, 60°C x 30s to 90s for 45 cycles as described previously (Yang et al., 2015). The primers specific for bacterial phyla Bacteroidetes and Firmicutes and a pair of universal primers are shown in Table 1. These primers were adopted from the published studies elsewhere (Bacchetti De Gregoris et al., 2011; Yang et al., 2015).

**Table 1:** The primers specific for bacterial phyla Bacteroidetes and Firmicutes and a pair of universal primers.

Firmicutes F	GGAGYATGTGGTTTAATTCGAAGCA
Firmicutes R	AGCTGACGACAACCATGCAC
Bacteroidetes F	GTTTAATTCGATGATACGCGAG
Bacteroidetes R	TTAASCCGACACCTCACGG
Universal F	AAACTCAAAKGAATTGACGG
Universal R	CTCACRRCACGAGCTGAC

### Statistics analyses

The number of live organisms expressed as IFUs, Log10 IFUs, 16S rRNA gene copies. The chlamydial genome copies and hydrosalpinx scores were compared between groups using Wilcoxon rank sum test. The incidences of hydrosalpinx between groups were evaluated by Fisher’s Exact Probability Test.^1^

Correlations of chlamydial pathogenicity in the upper genital tract with chlamydial colonization in different tissues were analyzed by calculating Spearman Rank-Order Correlation Coefficients.^2^ Furthermore, the Significance of the Difference Between Two Correlation Coefficients was calculated.^3^

## Results

### Oral ABX treatment significantly increases the susceptibility of hydrosalpinx induction by *Chlamydia muridarum* intravaginal infection in DBA2/J mice

**Figure 1:**
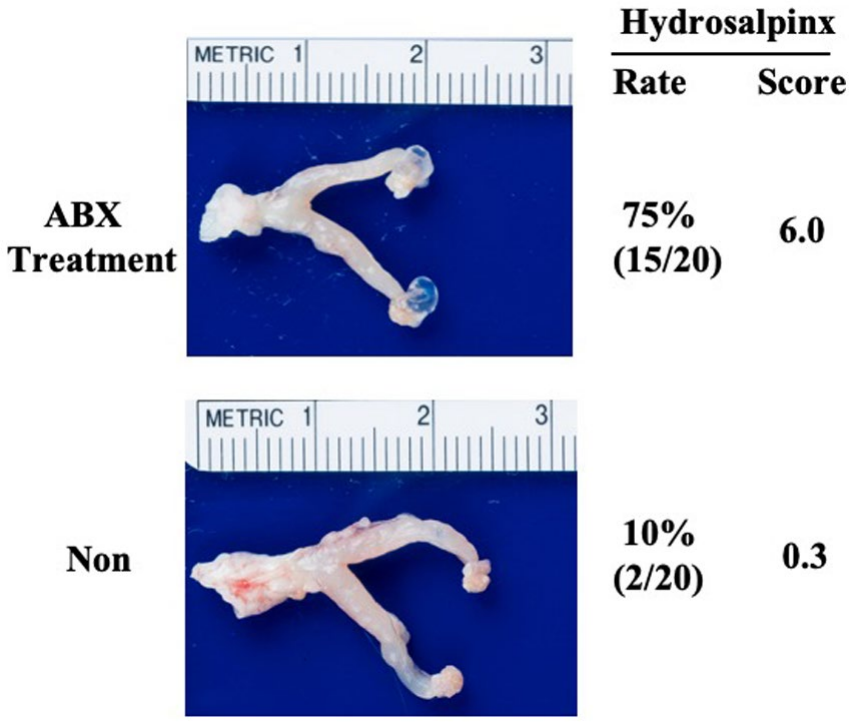
Oral ABX treatment significantly increases the susceptibility of hydrosalpinx induction by *C. muridarum* intravaginal infection in DBA2/J mice. Female DBA2/J mice without (Non, *n* = 20) or with (ABX, *n* = 20) treatment with vancomycin and gentamicin cocktail (ABX) for 21 days were intravaginally inoculated with a live *C. muridarum* clone (G13.32.1). The oral ABX was maintained in drinking water throughout the experiment. On day 70 after the intravaginal inoculation, all mice were sacrificed for evaluating genital tract pathology. Hydrosalpinx from each mouse was evaluated macroscopically under naked eye as described in the Materials and Methods. One representative genital tract image from each group was shown with the vagina on the left and oviduct/ovary on the right corresponding hydrosalpinx scores marked. Data came from 3 independent experiments. Note that ABX treatment significantly increased pathology rate and severity macroscopically. Fisher’s Exact for comparing rates and Wilcoxon rank sum for scores.

The previous study has showed that DBA2/J mice are naturally resistant to hydrosalpinx induction by intravaginal infection with *C. muridarum* (Chen et al., 2014). Here we used an oral administration of membrane-impermeable antibiotics vancomycin and gentamicin cocktail to induce gut dysbiosis in DBA2/J mice. As shown in Figure 1, female DBA2/J mice without the ABX treatment remained highly resistant to hydrosalpinx induction by *C. muridarum*, and the hydrosalpinx incidence rate was only 10% and a mean severity score was ~0.3. Only two out of 20 mice developed minimal levels of hydrosalpinx in 3 independent experiments. The lack of oviduct pathology was confirmed by microscopic evaluation of oviduct dilation and inflammatory infiltration. However, in the group of ABX treatment, the hydrosalpinx incidence increased to >70% with a mean severity score of ~6. A total of 15 out of 20 mice treated with the oral ABX cocktail developed significant hydrosalpinx. The evidence of significant oviduct dilation and tubal inflammatory infiltration was microscopically confirmed in these mice. As a result, we have demonstrated that oral administration with vancomycin and gentamicin is sufficient to promote chlamydial pathogenicity in the upper genital tract of DBA2/J mice.

### Oral ABX treatment did not alter chlamydial infection or microbiota in the genital tract

**Figure 2:**
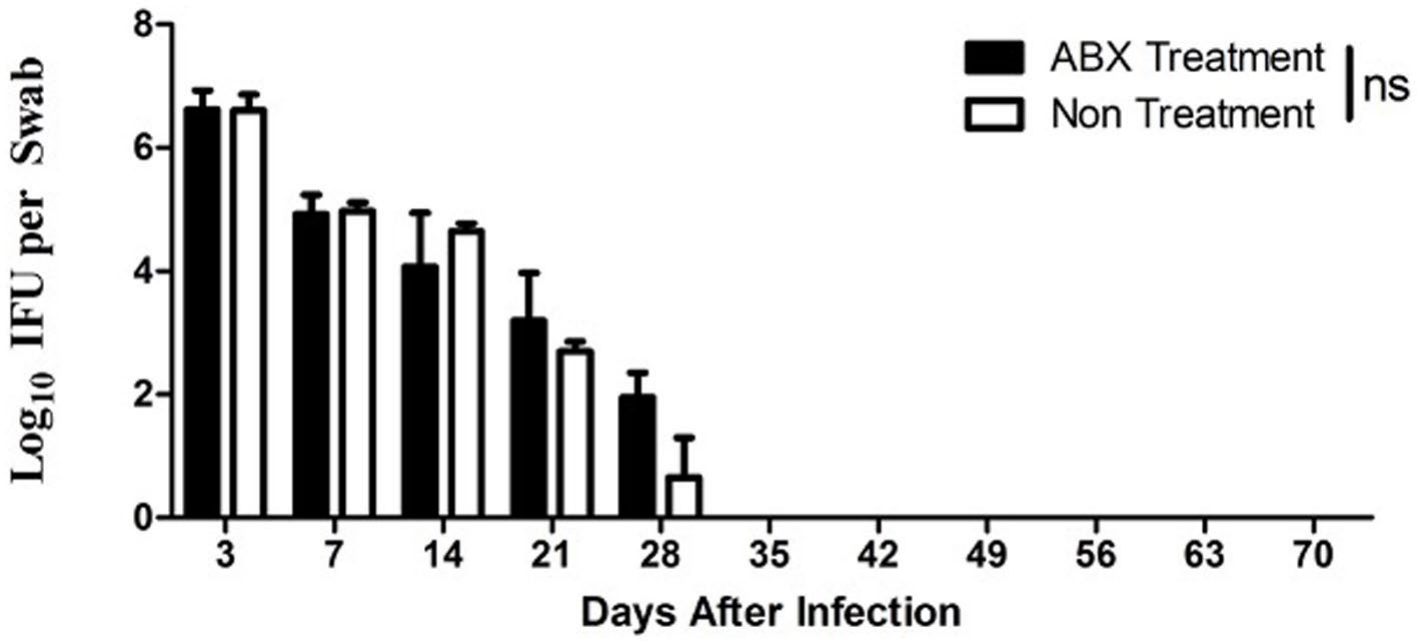
Oral ABX treatment did not alter chlamydial infection in the genital tract. Female DBA2/J mice without (Non, *n* = 20) or with (ABX, *n* = 20), oral antibiotics treatment for 21 days was intravaginally infected with *C. muridarum* as described in Figure 1 legend. Live organism shedding was monitored from vaginal swabs on days 3, 7 and weekly thereafter (up to day 70) as shown along X-axis. Both live organisms recovered (Log10 IFUs per swab) were shown along *Y*-axis. Note that significant shedding was similarly detected in all mice up to day 28 after infection regardless of the oral antibiotics’ treatment (*p* > 0.05, Wilcoxon for comparing area-under-curve).

**Figure 3:**
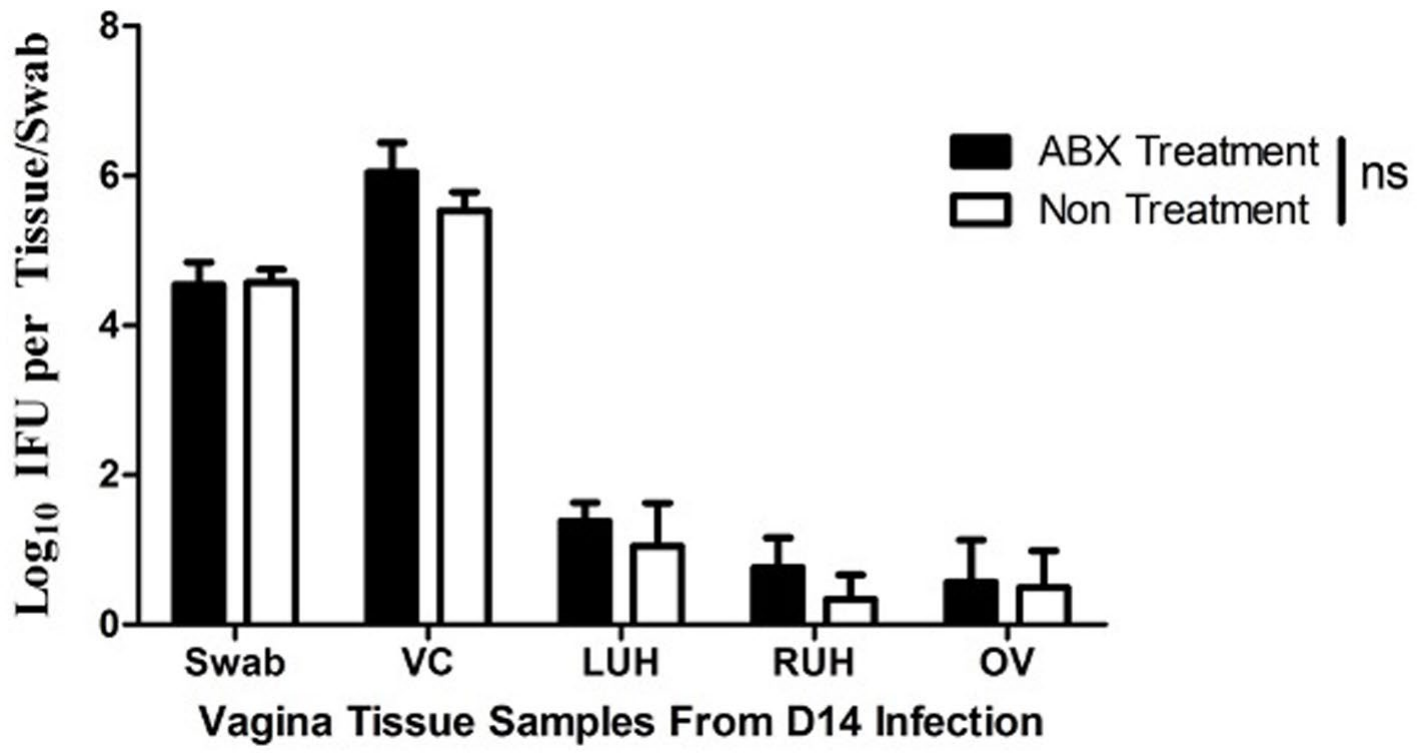
Oral antibiotics treatment fails to alter *C. muridarum* ascension to the upper genital tract. Female DBA2/J mice without (Non, *n* = 5) or with (ABX, *n* = 5) oral antibiotics treatment for 21 days were intravaginally inoculated with *C. muridarum.* The oral ABX was maintained in drinking water throughout the experiment. On day 14 after the intravaginal inoculation, all mice were sacrificed for titrating live organisms recovered from different regions of the genital tract including vagina/cervix (VC), left uterine horn (LU), right uterine horn (RU) and oviduct/ovary (RO) as shown along X-axis. The live organisms recovered were expressed as Log10 IFUs as shown along *Y*-axis. Note that live *C. muridarum* organisms were similarly detected in uterine horn and oviduct tissues of all mice regardless of the oral antibiotics’ treatment (*p* > 0.05, Wilcoxon for comparing IFUs from individual tissues between the two groups of mice).

It has been shown that the altered microbiota and enhanced chlamydial infection may impact the chlamydial pathogenicity (Chen et al., 2014; Ziklo et al., 2016). We evaluated the chlamydial infection or microbiota in the mice with or without ABX treatment. As shown in Figure 2, significant live organism shedding was similarly detected in all mice up to day 28 after the intravaginal infection regardless of the oral ABX treatment. The level of shedding and number of mice remaining positive for live organisms were both similar between mice with or without ABX treatment. Since the live organism shedding could only reflect chlamydial infections in the lower genital tract, we further tested the chlamydial infection by tissue homogenization of upper genital tract (Figure 3). On day 14 after intravaginal inoculation, the day of infection reaches the highest level, the mice were sacrificed to evaluate the live organism distribution across the whole genital tract. We recovered high levels of live chlamydial organisms from both sides of uterine horns and oviducts regardless of the oral ABX treatment. The oral ABX cocktail did not affect genital chlamydial ascension.

**Figure 4:**
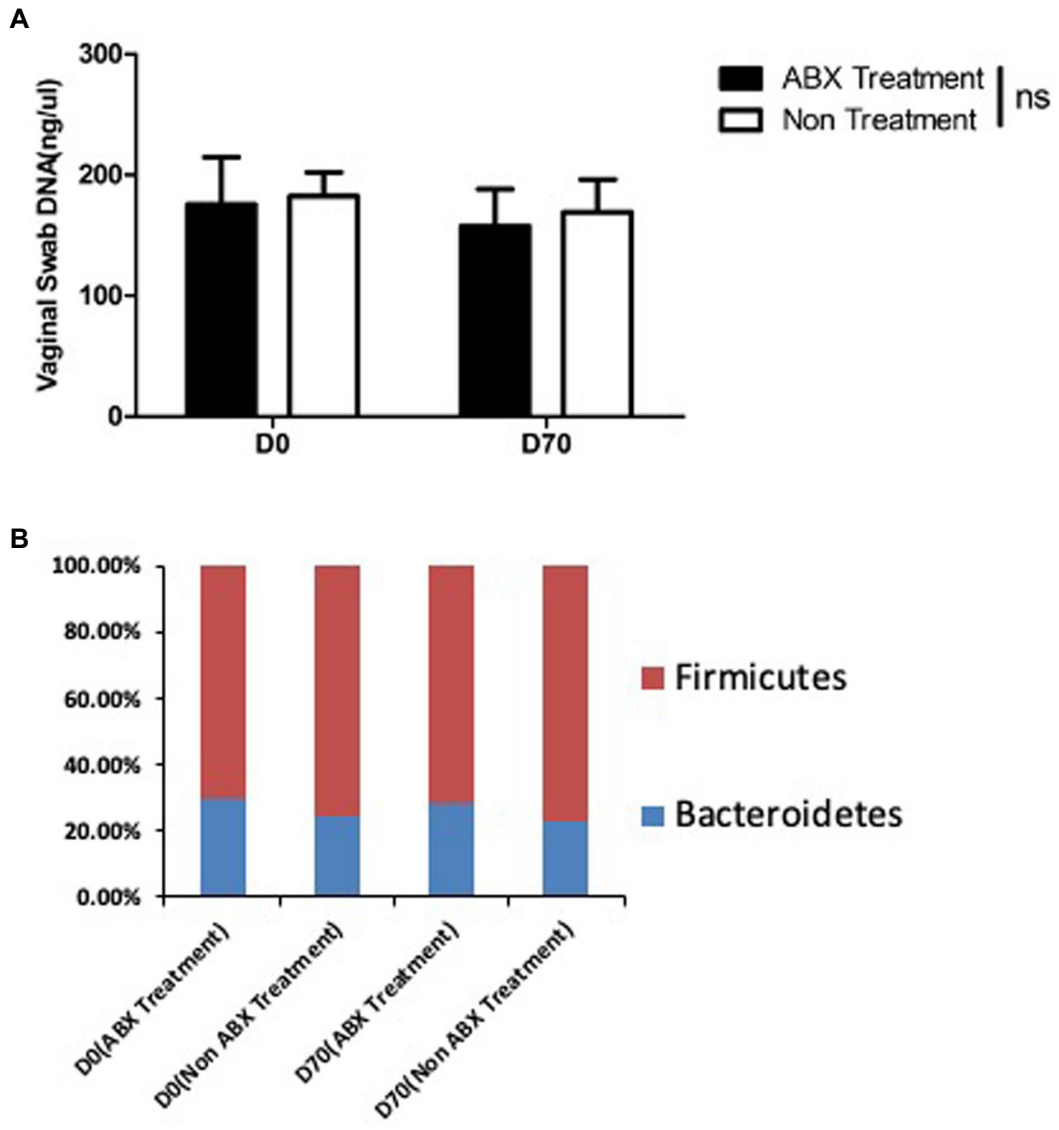
Oral antibiotics treatment fails to alter vaginal microbiota. Female DBA2/J mice were treated without (Non, *n* = 20) or with (ABX, *n* = 20) oral antibiotics for 21 days (D0 or prior to infection) or 91 days (D70, 70 days after infection) as described in Figure 1 legend. Vaginal swabs were taken for extracting DNA (panel **A**) and detecting the relative abundance of 16S rRNA gene from bacterial phyla bacteroidetes (blue, panel **B**) and firmicutes (red, panel **B**) using. Neither the vaginal DNA amount nor the relative abundance of bacteroidetes and firmicutes in the vaginal swabs was different between mice with or without the oral antibiotics treatment for 21 or 91 days (*p* > 0.05, Wilcoxon).

To further evaluate the effect of the oral antibiotics on genital microbiota, we compared the microbiota in vaginal swabs between mice with or without oral ABX treatment (Figure 4). We found that the amounts of DNA were similar from vaginal swabs regardless of the oral ABX treatment. More importantly, when 16S rRNA gene copy was quantitated using qPCR with primers targeting bacterial phyla bacteroidetes and firmicutes, the relative abundance of these two phyla was similar between mice with or without oral ABX treatment for 21 days (prior to infection) or 91 days (70 days after chlamydial infection). Thus, the oral ABX used in the current study did not significantly alter the microbiota in the genital tract.

### Oral antibiotics treatment induces dysbiosis without affecting *Chlamydia muridarum* colonization in the GI tract

**Figure 5:**
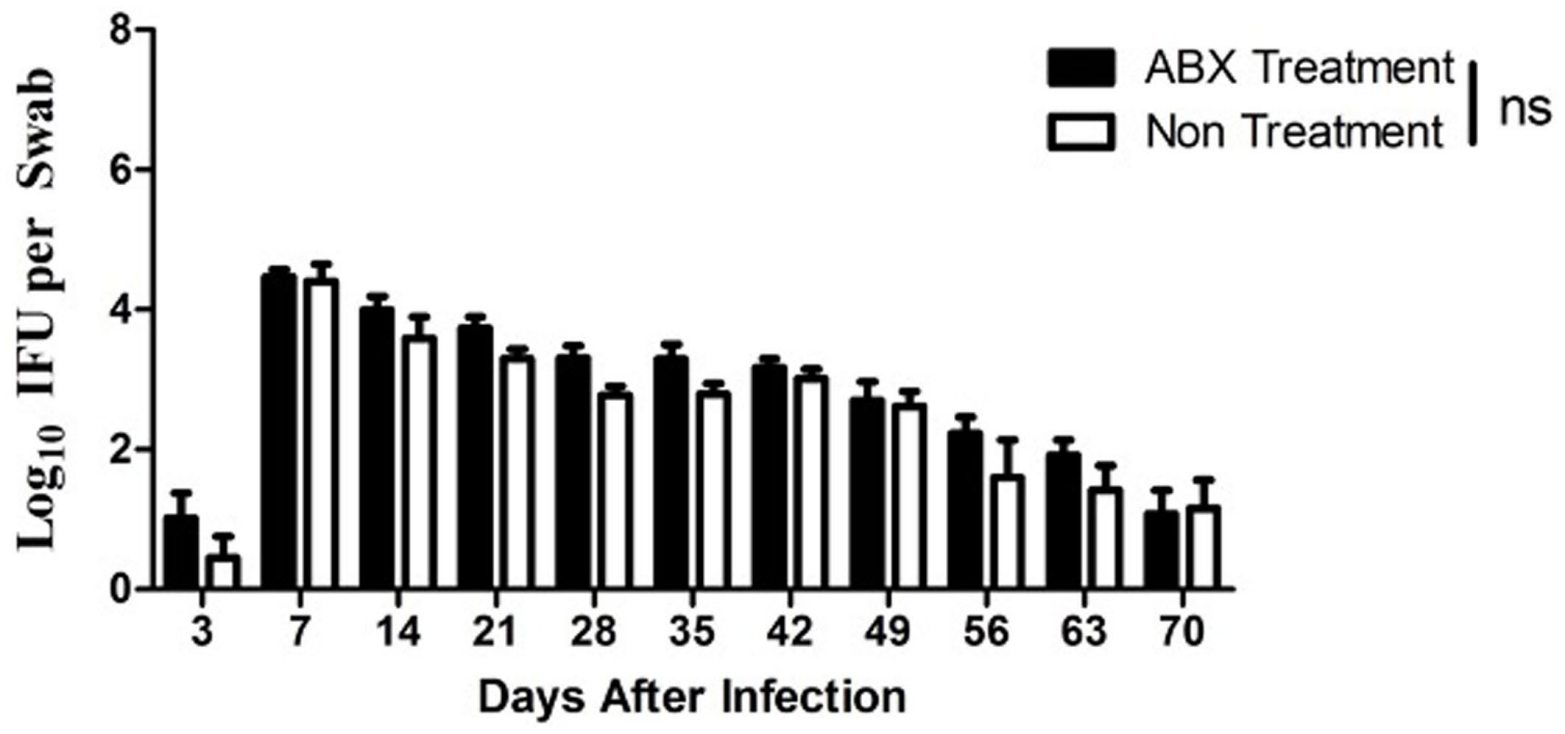
Oral antibiotics treatment did not alter the *C. muridarum* colonization in the gut. Female DBA2/J mice without (Non, *n* = 20) or with (ABX, *n* = 29) oral antibiotics treatment was intravaginally infected with *C. muridarum* as described in Figure 1 legend. Live organism shedding was monitored from rectal swabs on days 3, 7 and weekly thereafter as shown along *X*-axis. The live organisms recovered (Log10 IFUs per swab) were shown along Y axis. Note that significant shedding was similarly detected in all mice throughout the experiment regardless of the oral antibiotics treatment (*p* > 0.05, Wilcoxon for comparing area under curve).

**Figure 6:**
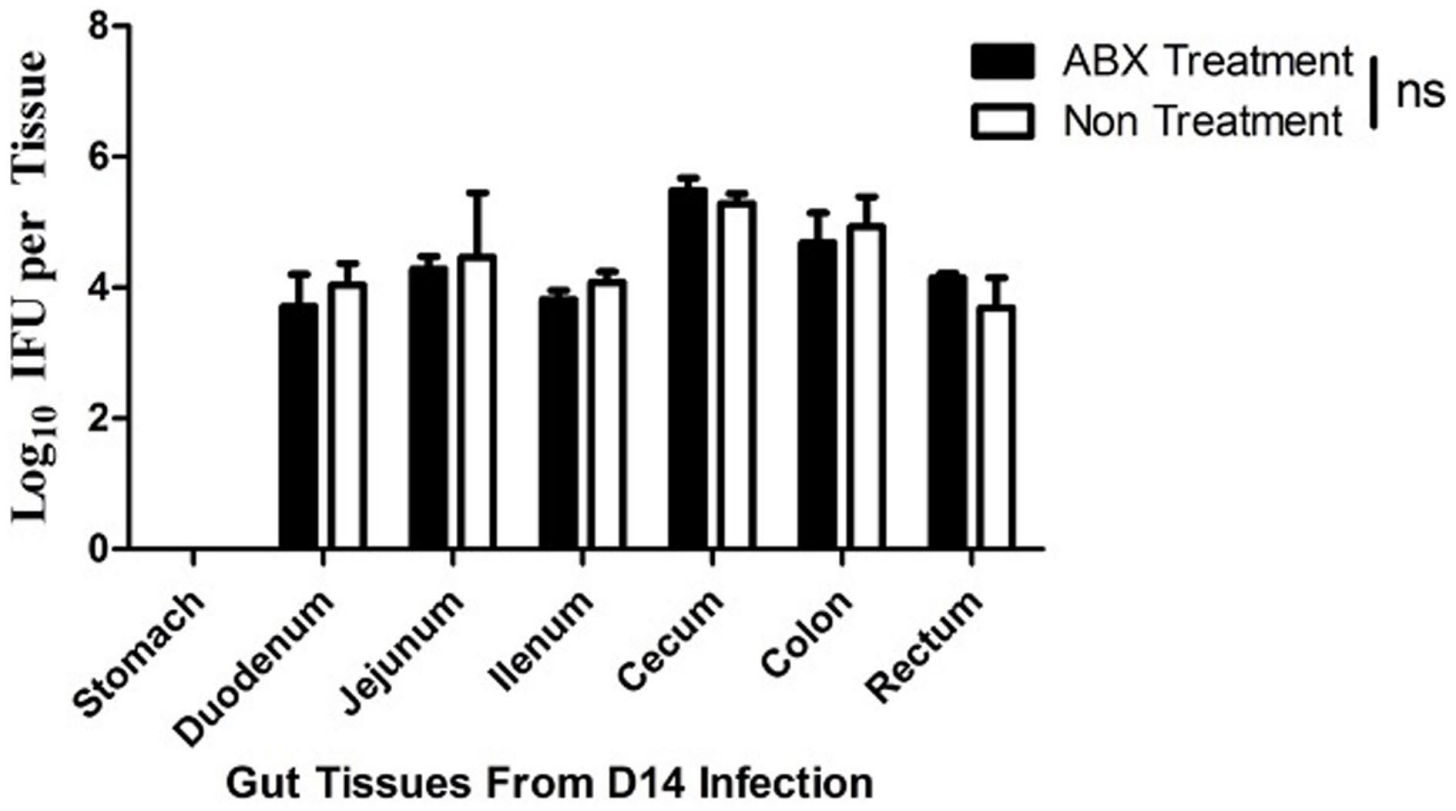
Oral antibiotics treatment fails to alter *C. muridarum* distribution in the mouse gastrointestinal tract. Female DBA2/J mice without (Non, *n* = 5) or with (ABX) oral antibiotics treatment for 21 days were intravaginally inoculated with *C. muridarum* as described in Figure 3 legend. On day 14 after the intravaginal inoculation, all mice were sacrificed for titrating live organisms recovered from different regions of the gastrointestinal tract including stomach, duodenum, jejunum, ilium, cecum, colon and rectum as shown along *X*-axis. The live organisms recovered were expressed as Log10 IFUs as shown along *Y*-axis. Note that live *C. muridarum* organisms were similarly detected in different regions of the gastrointestinal tracts from all mice regardless of the oral antibiotics treatment (*p* > 0.05, Wilcoxon for comparing IFUs from individual tissues between the two groups of mice).

**Figure 7:**
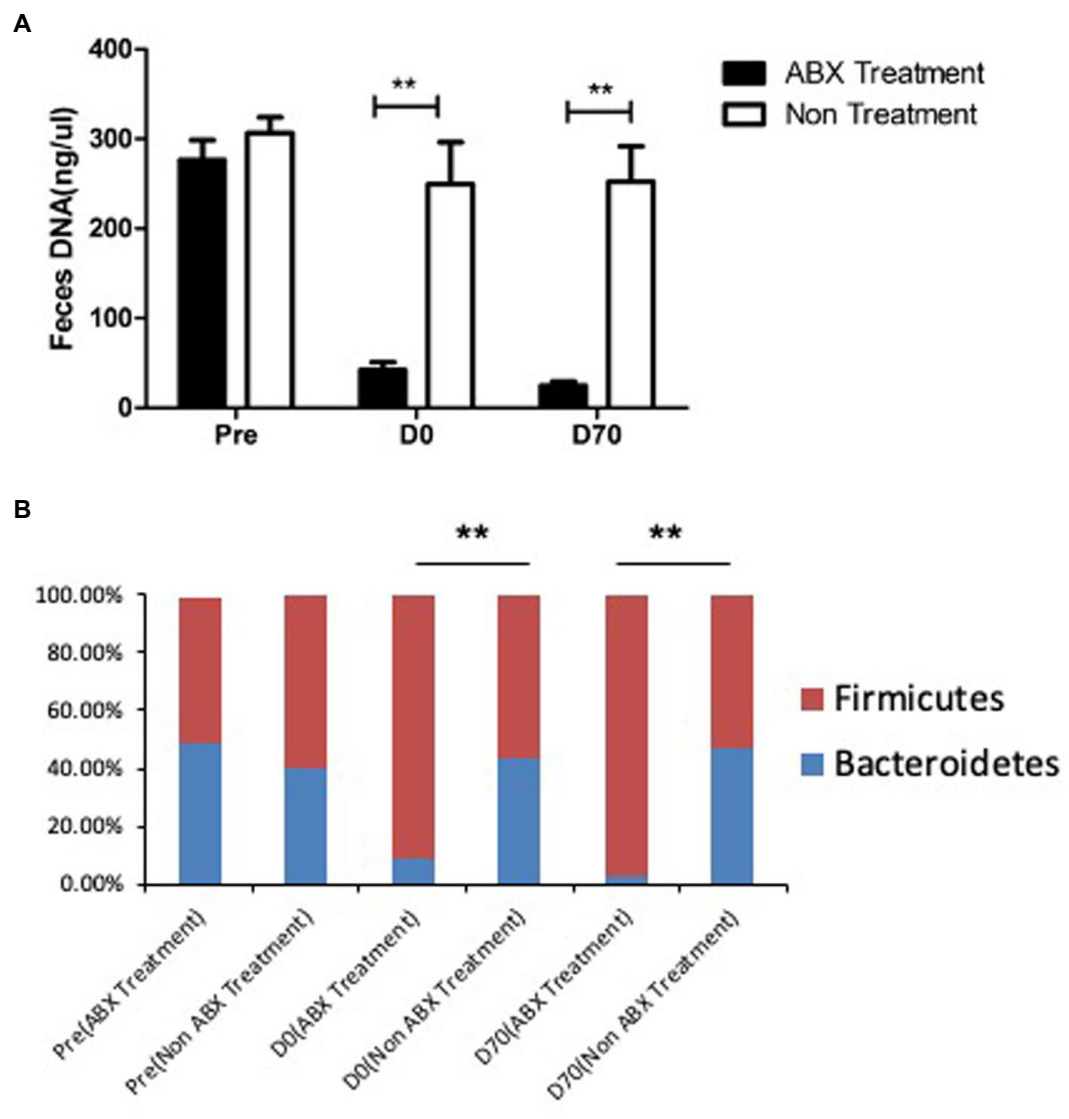
The fecal microbiota were changed sigificantly by oral antibiotics treatment. Female DBA2/J mice were treated without (Non, *n* = 20) or with (ABX, *n* = 20) oral antibiotics for 21 days as described in Figure 1 legend. Fecal samples were taken from mice prior to the oral antibiotics treatment (Pre), 21 days after the treatment but before chlamydial infection (D0) and 70 days after chlamydial infection (D70) for extracting DNA (panel **A**) and detecting the relative abundance of 16S rRNA gene from bacterial phyla bacteroidetes (blue, panel **B**) and firmicutes (red, panel **B**) using qPCR. Please note that both fecal DNA amount and relative abundance of bacteroidetes and firmicutes in the fecal samples were significantly different between mice with or without the oral antibiotics treatment for 21 days (**p < 0.01, Wilcoxon).

Since genital *C. muridarum* has been shown to spread to and colonize in the GI tract (Zhang et al., 2015) and the GI*C. muridarum* has been proposed to promote chlamydial pathogenicity in the upper genital tract (Zhong, 2018), we next evaluated the effect of the oral ABX on the chlamydial colonization (Figure 5) and distribution (Figure 6) in the GI tract. We found that the oral ABX cocktail did not affect the overall colonization of *C. muridarum* in the GI tracts up to 70 days after intravaginal infection. Mice with or without oral ABX treatment maintained relatively steady and similar infection time courses. On day 14 after intravaginal *C. muridarum* infection, some mice were sacrificed for evaluating the distribution of *C. muridarum*, we found that similar levels of live *C. muridarum* organisms were recovered from different gut tissues of all these mice regardless of the oral ABX treatment. Thus, chlamydial colonization and distribution in the GItract were not significantly altered by the oral ABX treatment. We then compared the fecal microbiota between mice with or without oral ABX treatment (Figure 7). We found that 21 days of ABX treatment in mice result in significant dysbiosis. The dysbiosis was maintained till the end of the experiments (up to 70 days after infection). The total fecal DNA of oral ABX-treated mice was significantly reduced, suggesting that the oral ABX treatment significantly reduced the number of bacteria in the gut. The reduced amount of fecal DNA reflected the reduction in the bacteria burden, while whether the bacterial composition or diversity has been changed is not clear. We then monitored the composition of bacterial phyla and we found significant alteration of bacterial composition with decreased bacteroidetes but increased firmicutes.

### Oral ABX-related hydrosalpinx correlates with GI microbiota profile

**Figure 8:**
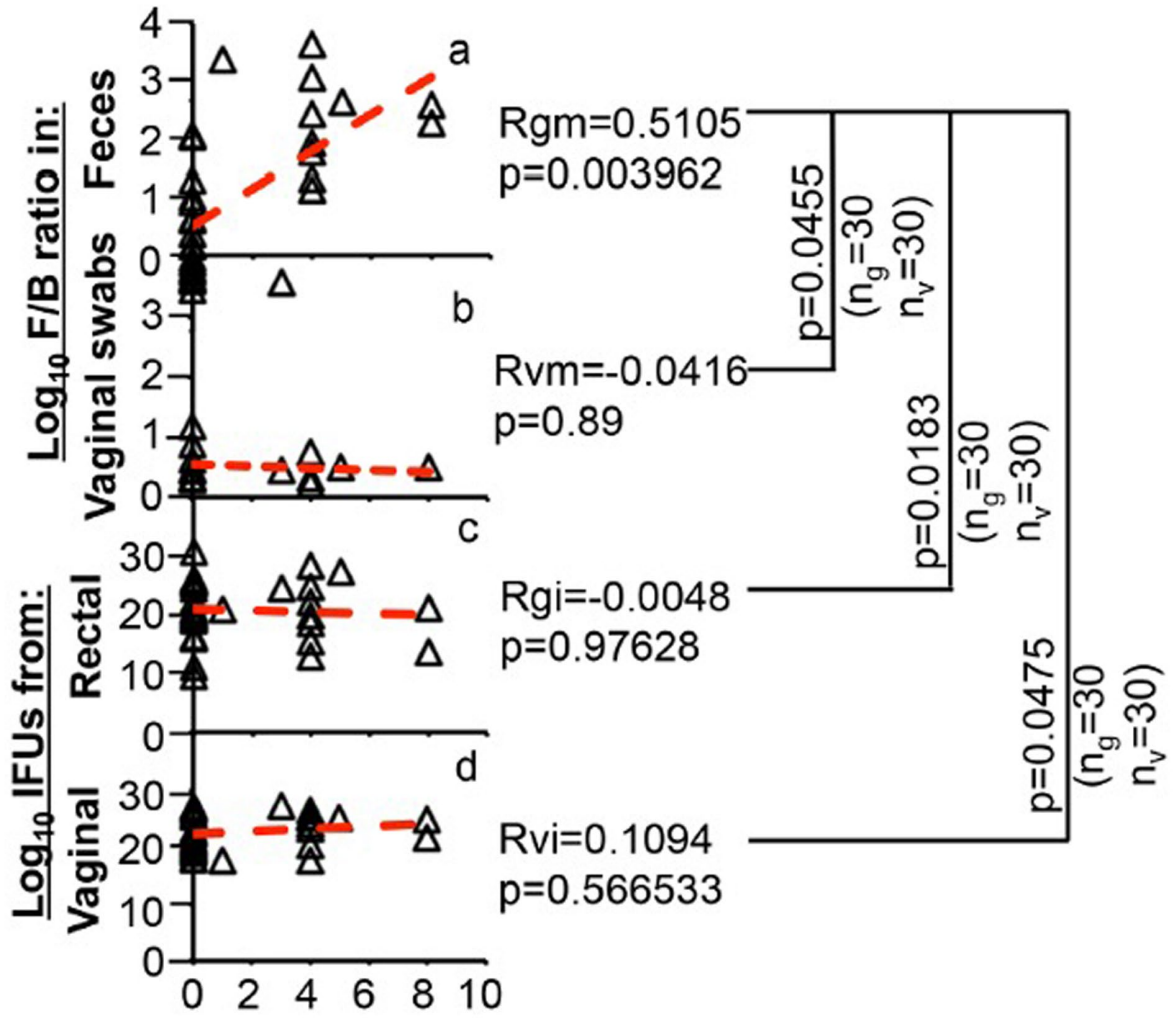
Correlation of oral antibiotics-enhanced hydrosalpinx with fecal microbiota but not vaginal microbiota or *C. muridarum* shedding from either genital or gastrointestinal tract. The hydrosalpinx score from each DBA2/J mouse as described in Figure 1 legend (*X*-axis) was used to correlate with microbiota profile expressed as Log10 F/B ratios obtained from feces (Y-axis, panel a) or vaginal swabs (*Y*-axis, b) or live organism shedding expressed as Log10 IFUs (total IFU from swabs collected from a given mouse over time, *Y*-axis) from rectal (c) or vaginal (d) swabs using the Spearman’s correlation formula as described in the materials and method section. The total IFU from each mouse was obtained by adding IFUs from all time points observed. The correlation co-efficient between the hydrosalpinx scores and gut (or feces) F/B ratios was defined as Rgm, which is 0.5105 with a *p* value of 0.0039, the correlation co-efficient between the hydrosalpinx scores and vaginal F/B ratios was Rvm = −0.0416 with a *p* = 0.89 (two tails), or rectal IFUs (Rgi = 0.0048, *p* = 0.9763) or vaginal IFUs (Rvi = 0.1094, *p* = 0.5665). A positive correlation was only identified between the hydrosalpinx scores and rectal F/B ratios, which was significantly stronger than those between hydrosalpinx and rectal F/B ratio or rectal or vaginal IFUs (*p* = 0.0455, 0.0183, 0.0475 respectively).

The oral ABX treatment promoted hydrosalpinx development and induced gut dysbiosis without altering microbiota in the genital tract. The GI and genital chlamydial colonization wasn’t influenced by ABX treatment either. We used a Spearman’s correlation analysis to mathematically define the correlation (Figure 8) between the hydrosalpinx scores and gut dysbiosis. It is shown that the hydrosalpinx scores positively correlated with gut dysbiosis scores expressed as Log10 F/B ratios obtained from feces. However, the hydrosalpinx scores did not correlate with Log10 F/B ratios from vaginal swabs or live chlamydial organism shedding (Log10 IFUs) from rectal or vaginal swabs.

The correlation co-efficient between the hydrosalpinx scores and gut dysbiosis was 0.51 (with a *p* value of 0.0039) and the correlation co-efficient between the hydrosalpinx scores and vaginal F/B ratios or rectal IFUs vaginal IFUs were −0.0416 (*p* = 0.89), 0.0048 (*p* = 0.9763), and 0.1094 (*p* = 0.5665) respectively (Figure 8). The significant mathematical correlation was only identified between the hydrosalpinx scores and fecal F/B ratios. This correlation was significantly stronger than those between hydrosalpinx and vaginal F/B ratio or rectal or vaginal IFUs (*p* = 0.0455, 0.0183, 0.0475 respectively). As a result, we identified a significant association of gut dysbiosis with chlamydial pathogenicity in the upper genital tract.

## Discussion

In the current study, we used an oral ABX cocktail to induce gut dysbiosis in the DBA2/J mice with *C. muridarum* genital infection and we tried to demonstrate the role of gut dysbiosis in chlamydial pathogenicity in the upper genital tract with this model. The oral administration of membrane impermeable antibiotics vancomycin and gentamicin significantly enhanced hydrosalpinx development in DBA2/J mice with *C. muridarum* genital infection. The DBA2/J mice were known to be highly resistant to hydrosalpinx induction by gential *C. muridarum* infection, while the oral ABX treatment consistently enhanced hydrosalpinx development. This enhancement effect was demonstrated in 3 independent experiments. Firstly, the ABX treatment did not significantly alter either chlamydial infection in the genital tract, and it suggested that the oral ABX promoted hydrosalpinx through the mechanisms independent of the genital Chlamydia. Furthermore, the oral ABX treatment did not affect genital *C. muridarum* spreading to or colonizing in the GI tract, suggesting that the oral ABX treatment did not promote hydrosalpinx by increasing chlamydial colonization in GI tract either. Finally, the oral ABX caused severe dysbiosis in the GI tract, including reduction in fecal DNA and increase in ratios of firmicutes over bacteroidetes. The oral antibiotics- enhanced hydrosalpinx only correlated with dysbiosis in the gut but not genital tract. It is noteworthy to emphasize that although the Firmicutes to Bacteroidetes ratio in the gut has been considered as an indicator of dysbiosis (Stojanov et al., 2020; Kapoor et al., 2023), this metric alone may not sufficiently discriminate differences in the bacterial species composition. In addition, it may not provide insights into the changes in bacterial diversity and evenness, or the co-occurrence of bacterial networks associated with specific microbiota states as potential pathological biomarkers. Therefore, more advanced metagenomic approaches (Gupta et al., 2023; Szóstak et al., 2023), such as 16S rRNA sequencing or shotgun sequencing of all bacterial genomes, may be required to explicitly address these issues. Despite the limitations, our study provides a new direction for further understanding chlamydial pathogenicity.

The oral antibiotics used in many other gut microbiome studies often include neomycin, ampicillin and metronidazole in addiction to vancomycin or gentamicin (Kennedy et al., 2018). In order to selectively target gut lumenal bacteria, we used a cocktail consisting of only vancomycin and gentamicin since these two antibiotics cannot pass through gut epithelial barrier and fail to inhibit chlamydial growth (Ridgway et al., 1978; Dailloux et al., 1990). Indeed, we observed that the orally delivered vancomycin and gentamicin cocktail did not alter colonization of *C. muridarum* nor other bacteria in the genital tract. It failed to affect genital *C. muridarum* spreading to and colonizing in the GI tract as well. This selective targeting of gut lumenal bacteria by the oral ABX used in the current study has allowed us to associate the antibiotics-enhanced hydrosalpinx with gut dysbiosis. The next question is how the oral ABX-induced gut dysbiosis enhanced chlamydial pathogenicity in the upper genital tract. In recent years, gut microbiota has been associated with extra-gut pathologies (Tai et al., 2016; Shivaji, 2017). It is suggested that gut microbiota may even regulate fibrosis (Burke et al., 2017; Ho and Varga, 2017; Loomba et al., 2017). Since fibrosis is a key feature of chlamydial pathogenicity in the upper genital tract (Rodgers et al., 2010, 2011; Budrys et al., 2012; Chen et al., 2014; Lei et al., 2014; Liu et al., 2014; Yang et al., 2014; Zhong, 2017), we hypothesize that gut dysbiosis induced by the oral ABX in DBA2/J mice may promote profibrotic responses in the genital tract following chlamydial infection.

Normal microbiota is required for maintaining immunity to insults (Belkaid and Hand, 2014). Gut dysbiosis may skew the host immune responses toward Th2-dominant response that may favor tissue repairing and fibrosis. The increase in the ratio of Firmicutes over Bacteroidetes in gut microbiota has been associated with exacerbation of pathologic responses (Chen et al., 2011; Russell et al., 2012; Arrieta et al., 2014; Belkaid and Hand, 2014; Arrieta et al., 2015; Block et al., 2016; Brown et al., 2017; Budden et al., 2017; Ipci et al., 2017; Kang et al., 2017; Lin and Zhang, 2017; Samuelson et al., 2017; Shivaji, 2017; Wood et al., 2017). The increased ratio of Firmicutes over Bacteroidetes in gut microbiota can also be accompanied with increased segmental filamentous bacteria (SFB) that are known to induce Th17 (Gauguet et al., 2015; McAleer et al., 2016). Although Th17 is not as effective as Th1 in clearing chlamydial infection, Th17 cells have been shown to promote chlamydial pathogenicity in the upper genital tract (Moore-Connors et al., 2013). Thus, we assume that the oral ABX-induced dysbiosis may promote chlamydial induction of hydrosalpinx without significantly altering chlamydial infectivity. We will use the oral vancomycin and gentamicin treatment of DBA2/J mouse intravaginal infection model to testing this and other hypotheses in future studies.

### Limitations of the study

The current study has a limitation in that it utilized real-time quantitative PCR to target only total 16S rRNA belonging exclusively to the bacterial phyla Bacteroidetes and Firmicutes to investigate the gut and genital microbiota. The approach is inadequate for assessing bacterial taxonomic diversity and track shifts in bacterial community structure and organization, which could have diagnostic value as indicators of pathological conditions. More advanced metagenomic approaches, such as 16S rRNA sequencing or shotgun sequencing of all bacterial genomes, were not utilized in this study and are required in order to perform such analyses.

Additionally, the study solely focused on DBA/2J mice with oral antibiotic-induced dysbiosis as a model for investigating the association between gut microbiota and chlamydial genital pathogenicity. It remains unknown if these findings can be extrapolated to other chlamydia-resistant mice models.

## Data availability statement

The original contributions presented in the study are included in the article/supplementary material, further inquiries can be directed to the corresponding authors.

## Ethics statement

All of the animal studies were approved by the Ethics Committee of the Institute of Pasteur of Shanghai Chinese Academy of Sciences.

## Author contributions

QT, TZ, and LW: conceptualization. QT, TZ, JM, and XS: methodology. QT, TZ, LW, and XS: investigation. QT, TZ, and LW: writing—original draft. QT and XS: writing—review and editing. TZ and QT: supervision. TZ and QT: funding acquisition. All authors contributed to the article and approved the submitted version.

## Funding

This research is supported by National Natural Science Foundation of China for the Youth (32100162) to QT and (32000138) to TZ.

## Conflict of interest

The authors declare that the research was conducted in the absence of any commercial or financial relationships that could be construed as a potential conflict of interest.

The reviewer ZZ declared a shared parent affiliation with the authors LW, XS to the handling editor at the time of review.

## Publisher’s note

All claims expressed in this article are solely those of the authors and do not necessarily represent those of their affiliated organizations, or those of the publisher, the editors and the reviewers. Any product that may be evaluated in this article, or claim that may be made by its manufacturer, is not guaranteed or endorsed by the publisher.

## Footnotes

^1^ http://vassarstats.net/tab2x2.html

^2^ http://vassarstats.net/corr_rank.html

^3^ http://vassarstats.net/rdiff.html
